# Combined oblique lateral interbody fusion and spinal endoscopy (Endo-OLIF) for degenerative lumbar disease: early clinical outcomes of a novel minimally invasive approach

**DOI:** 10.3389/fsurg.2026.1901616

**Published:** 2026-07-23

**Authors:** Kuan Lu, Can Cao, Yusong Guo, Xiaohua Li, Zhaohui Li, Chuang Liu, Jingchao Wei, Shangju Gao, Wenyi Li

**Affiliations:** Department of Orthopedics, Hebei General Hospital, Shijiazhuang, Hebei, China

**Keywords:** clinical outcomes, degenerative lumbar disease, minimally invasive surgery, oblique lumbar interbody fusion, spinal endoscopy

## Abstract

**Background:**

The combination of oblique lumbar interbody fusion (OLIF) and spinal endoscopy presents a promising minimally invasive approach for treating degenerative lumbar diseases, leveraging the strengths of both techniques to optimize clinical outcomes. We term this combined technique Endo-OLIF (endoscopy-assisted oblique lumbar interbody fusion).

**Objective:**

This study aimed to evaluate the early clinical efficacy and technical nuances of Endo-OLIF in the management of degenerative lumbar conditions.

**Methods:**

A retrospective analysis was conducted on 29 patients who underwent Endo-OLIF for degenerative lumbar diseases at our institution between February 2022 and November 2023. Inclusion criteria specified treatment of no more than two contiguous levels between L2 and L5. Patients with left-sided disc herniation or predominant left-sided stenosis were excluded due to technical considerations of the endoscopic working trajectory. Clinical outcomes were assessed using the visual analogue scale (VAS) for pain and the Oswestry Disability Index (ODI) for functional disability. The mean follow-up duration was 15.6 months (range 12–22 months).

**Results:**

All procedures were successfully completed without intraoperative complications such as nerve root injury or dural sac injury. Postoperatively, two patients (6.9%) experienced transient ipsilateral thigh numbness or hip flexion weakness, which we attribute to intraoperative retraction and blunt stimulation of the lumbar plexus; both resolved completely within 6 weeks without specific intervention. Postoperative imaging confirmed satisfactory interbody fusion and complete removal of herniated disc material. Significant improvements in VAS and ODI scores were observed at 3 months postoperatively and maintained at final follow-up (mean 15.6 months) (*P* < 0.05 for all comparisons). The mean VAS scores for back pain improved from 7.1 ± 1.3 preoperatively to 1.6 ± 0.7 at final follow-up; leg pain VAS improved from 6.3 ± 1.2 to 1.3 ± 0.5; ODI improved from 66.8 ± 9.7 to 12.6 ± 7.1. Patients reported high satisfaction levels, short recovery times, and no serious postoperative complications.

**Conclusion:**

Endo-OLIF capitalizes on the advantages of both minimally invasive approaches: OLIF preserves the posterior ligamentous complex and avoids nerve root irritation, while spinal endoscopy allows direct decompression by removing herniated disc material. This combined approach demonstrates favorable short-term outcomes with significant improvements in pain and functional disability scores. However, long-term efficacy requires further follow-up studies.

## Introduction

Lumbar degenerative disease (LDD), characterized by intervertebral disc degeneration, lumbar spinal stenosis, and spondylolisthesis, is a prevalent spinal disorder that constitutes a leading cause of disability and impaired quality of life among aging populations (1). The pathogenesis of LDD involves age-related degeneration of intervertebral discs, which is a major cause of low back pain worldwide. Additionally, facet joint hypertrophy and ligamentum flavum thickening contribute to anatomical narrowing of the spinal canal and lateral recess, ultimately leading to nerve root compression and chronic low back pain (2). Traditional surgical treatments, such as posterior lumbar interbody fusion (PLIF) and transforaminal lumbar interbody fusion (TLIF), have been widely used to achieve neural decompression and spinal stabilization. However, these approaches often require extensive dissection of paraspinal muscles, which may result in prolonged recovery, increased blood loss, and postoperative complications such as muscle denervation and failed back surgery syndrome (3). These limitations have driven the development of minimally invasive techniques to improve clinical outcomes.

Oblique lumbar interbody fusion (OLIF) has emerged as a promising minimally invasive technique for treating LDD. Initially introduced in 2012, OLIF utilizes a retroperitoneal approach through the anatomical window between the psoas muscle and the aorta, avoiding direct injury to the lumbar plexus and posterior spinal structures (4). This approach allows for effective disc space preparation, restoration of lumbar lordosis, and placement of a large interbody cage, which contributes to higher fusion rates (5). Compared with traditional PLIF or TLIF, OLIF demonstrates advantages such as reduced intraoperative blood loss, shorter hospital stays, and lower rates of postoperative complications (6). These benefits position OLIF as an attractive alternative for patients with LDD. However, its therapeutic mechanism relies on indirect decompression via disc space distraction rather than direct intraspinal decompression, which limits its applicability in severe spinal stenosis.

Spinal endoscopy has transformed the surgical management of LDD by enabling direct visualization and targeted decompression of neural structures. This technique allows for precise removal of herniated disc material and decompression of nerve roots with minimal tissue disruption (7). Studies have shown that spinal endoscopy results in reduced postoperative pain, faster recovery, and lower complication rates compared with open surgical techniques (8, 9). Additionally, the ability to perform spinal endoscopy under local anesthesia further enhances its appeal for high-risk patients who are unsuitable for general anesthesia (10). These advantages position spinal endoscopy as a valuable tool in the minimally invasive treatment of LDD.

Endo-OLIF (endoscopy-assisted oblique lumbar interbody fusion) achieves direct intraspinal decompression by incorporating endoscopic techniques during the OLIF procedure. Despite the individual benefits of OLIF and spinal endoscopy, there is limited research on the combined application of these techniques for treating LDD. The technical details, feasibility, and early clinical outcomes of this combined approach remain poorly understood (11). Furthermore, there is a lack of standardized protocols for Endo-OLIF, particularly in terms of patient selection, surgical planning, and postoperative management (12). This study aimed to address these gaps by evaluating the early clinical efficacy and technical considerations of Endo-OLIF in the treatment of LDD. By providing detailed insights into this innovative approach, our findings may contribute to optimizing minimally invasive strategies for managing lumbar degenerative conditions.

## Materials and methods

### Study population

This retrospective study was approved by the Ethics Committee of Hebei General Hospital [Approval No. (2026-LW-201)]. Written informed consent was waived by the ethics committee due to the retrospective nature of the study and the use of anonymized data. Consecutive patients who underwent Endo-OLIF for LDD between February 2022 and November 2023 were screened for eligibility. Demographic data were extracted from medical records, including age, sex, and body mass index (BMI). The mean follow-up duration was 15.6 months (range 12–22 months).

The inclusion criteria were as follows: (1) LDD diagnosed by both clinical and radiological findings, including lumbar disc herniation (LDH) with spinal stenosis, LDH with spondylolisthesis (Meyerding grade I–II), and LDH with degenerative lumbar scoliosis; (2) patients received Endo-OLIF at no more than two contiguous levels between L2 and L5 (i.e., single-level or two-level fusion); (3) complete clinical and radiological data, including x-rays, CT, and MRI studies of the lumbar spine; (4) a follow-up period of at least 12 months. Patients were excluded based on the following criteria: (1) presence of lumbar fractures, infections, neoplastic conditions, or severe osteoporosis (T-score ≤ −3.0); (2) history of previous lumbar spinal surgery; (3) previous major abdominal procedures that could compromise the retroperitoneal approach. (4) imaging findings indicating left-sided disc herniation or predominant left-sided spinal stenosis at the responsible level.

### Surgical procedure

The patient was positioned in the right lateral decubitus position on a radiolucent operating table under general anesthesia. Adequate padding was applied to pressure points, including the arms and knees, to prevent neurovascular complications. The surgeon stood on the ventral side of the patient, with the C-arm positioned on the opposite side for fluoroscopic guidance. The surgical table was adjusted to ensure true lateral imaging of the lumbar spine, particularly for multilevel cases. Under fluoroscopic guidance, the target intervertebral disc level was identified, and lines were drawn along the disc space and vertebral body margins. A 4–5 cm oblique skin incision was made approximately one-third of the distance from the iliac crest to the umbilicus. For multilevel cases, the incision was adjusted ventrally to intersect the midsection of the target levels. Blunt dissection was performed through the external oblique, internal oblique, and transversus abdominis muscles using a muscle-splitting technique. The retroperitoneal space was accessed, and the peritoneum was gently retracted anteriorly to expose the psoas muscle. The anterior border of the psoas muscle was identified and gently retracted dorsally to avoid injury to the lumbar plexus. Extraperitoneal fat, peritoneum, ureter, and abdominal contents were carefully retracted ventrally to protect these critical structures and further expose the surgical area. After adequately retracting the surrounding tissues, the target intervertebral disc and the adjacent superior and inferior vertebral bodies were clearly exposed.

Two guidewire pins were placed on the upper and lower vertebral bodies, respectively. These pins helped retract soft tissues, improved visualization of the surgical field, and served as anatomical landmarks for accurate lateral screw insertion during final fixation. Two retractors (ventral and dorsal) were positioned to maintain exposure without the need for a tubular retractor. Disc material was removed using curettes, shavers, and pituitary forceps. Meticulous endplate preparation was performed to preserve the bony endplates while ensuring adequate decortication for fusion.

A full-endoscopic system with a working channel was introduced through the OLIF corridor. The endoscope provided magnified visualization of the posterior disc space and neural structures. Migrated disc fragments, particularly those located posteriorly or within the epidural space, were removed using articulated endoscopic instruments. A tip-controlled high-speed burr was used to address posterior osteophytes and create additional working space. The posterior longitudinal ligament (PLL) was carefully resected to ensure complete neural decompression. The endpoint of decompression was confirmed by observing pulsatile movement of the dura mater and free flow of cerebrospinal fluid.

The contralateral annulus fibrosus was opened with a reamer, the intervertebral space was gradually expanded, and the disc space height was tested. Subsequently, an appropriately sized polyetheretherketone (PEEK) cage, packed with demineralized bone matrix (e.g., Grafton Putty, Medtronic), was inserted orthogonal to the disc space under fluoroscopic guidance. The cage position was confirmed using anteroposterior and lateral fluoroscopy. Using the guidewire pins as anatomical references, lateral screws were inserted into the vertebral bodies to enhance construct stability and prevent cage migration. The screws were placed under fluoroscopic guidance to ensure optimal trajectory and depth. The surgical site was irrigated thoroughly, and the retractors were removed. The abdominal muscles, subcutaneous tissue, and skin were closed in layers. A small drainage catheter was placed to prevent postoperative hematoma formation.

Postoperatively, the patient was monitored closely for neurological deficits and surgical complications. Early ambulation was encouraged, and a lumbar brace was prescribed for additional support during the initial recovery period.

### Clinical evaluation

The visual analogue scale (VAS) was used to evaluate low back pain and leg pain, where a score of 0 indicated no pain and 10 represented the worst pain. The Oswestry Disability Index (ODI) was used to evaluate functional disability. In addition, intraoperative parameters were recorded, including estimated blood loss (EBL), total operative time, and spinal endoscopic decompression time. Length of hospital stay (LOS) after surgery was recorded to evaluate postoperative recovery.

### Radiographic evaluation

**Figure 1:**
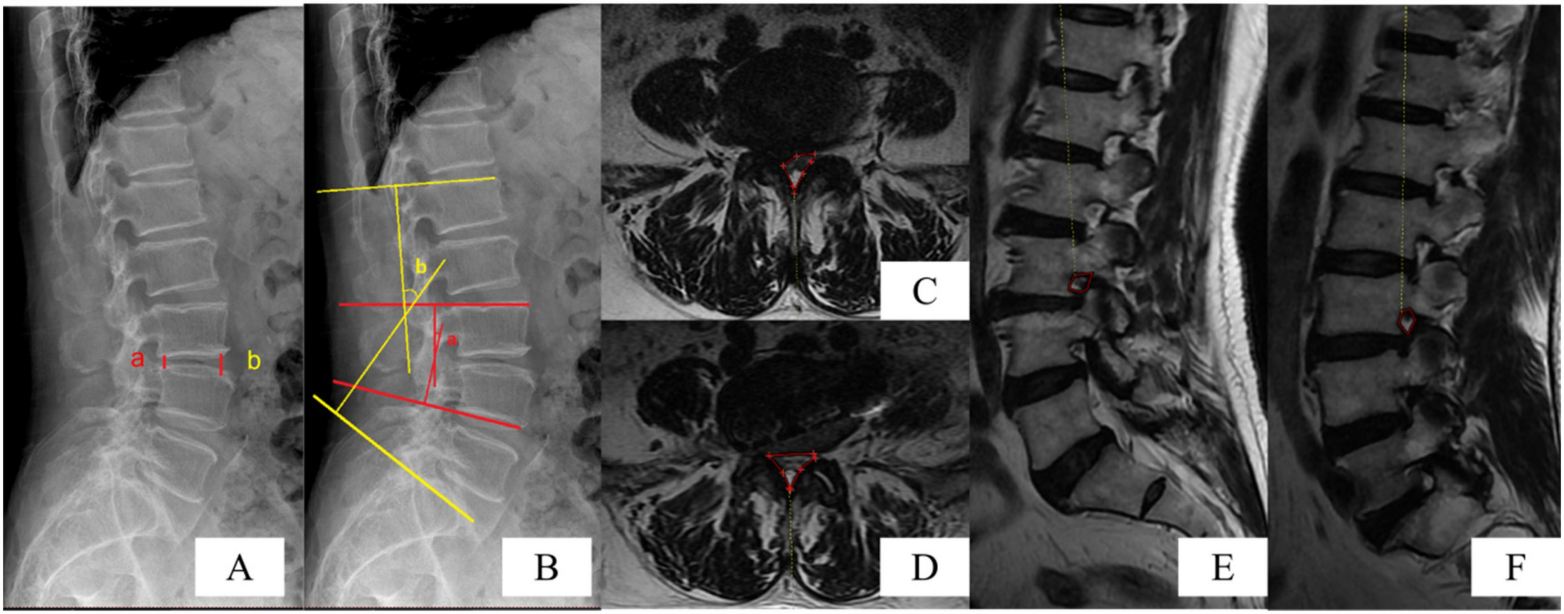
Quantitative measurements of radiographic parameters. **(A)** Measurement of intervertebral disc height (a: posterior disc height; b: anterior disc height). **(B)** Measurement of segmental lordotic angle **(a)** and global lumbar lordotic angle **(b) (C,D)** Preoperative **(C)** and postoperative **(D)** spinal canal area measurement on axial MRI. **(E,F)** Left **(E)** and right **(F)** intervertebral foramen area measurement on sagittal MRI.

Disc height (DH) was measured at the midpoint of the intervertebral space on lateral x-rays. Anterior vertebral height (AH) and posterior vertebral height (PH) of the fused segment were measured on lateral radiographs. DH (mm) was calculated using the formula: DH = (AH + PH)/2. Segmental lordosis angle and lumbar lordosis angle were calculated using the Cobb method. For lumbar spine MRI, spinal canal area was calculated on axial images at the level of maximal stenosis, and foraminal areas were measured on parasagittal MRI reconstructions (Figure 1).

### Statistical analysis

Statistical analysis was performed using SPSS version 26.0 (IBM Corp., Armonk, NY, USA). Continuous variables were expressed as mean ± standard deviation (SD) and compared using paired or independent t-tests. Categorical variables were presented as frequencies and percentages and analyzed using the chi-square test or Fisher's exact test. A *P*-value < 0.05 was considered statistically significant.

## Results

### Patient demographics and perioperative outcomes

A total of 29 patients (11 males, 18 females) met the inclusion criteria and were included in this analysis. The mean age was 60.2 ± 7.5 years (range 48–78 years), and the mean body mass index (BMI) was 25.8 ± 3.1 kg/m².

The cohort comprised three diagnostic categories: lumbar spinal stenosis with disc herniation (*n* = 15, 51.7%), lumbar spondylolisthesis (Meyerding grade I–II) with disc herniation (*n* = 12, 41.4%), and degenerative lumbar scoliosis with disc herniation (*n* = 2, 6.9%).

Regarding surgical levels: 27 patients (93.1%) underwent single-level Endo-OLIF (L2–L3: 4 cases, L3–L4: 7 cases, L4–L5: 16 cases), and 2 patients (6.9%) underwent two-level Endo-OLIF (L3–L5) for degenerative scoliosis-associated disc herniation. All two-level procedures met the inclusion criterion of ≤2 contiguous levels.

All procedures were successfully completed without conversion to open surgery or intraoperative complications such as dural tear, nerve root injury, or vascular injury. The mean estimated blood loss (EBL) was 108.7 ± 32.4 mL, and no patient required intraoperative blood transfusion. The mean total operative time was 123.5 ± 18.6 min, of which the spinal endoscopic decompression component accounted for 58.2 ± 10.3 min (47.1% of total operative time). The mean length of hospital stay (LOS) after surgery was 5.8 ± 1.7 days (range 3–10 days). No patient required readmission within 30 days postoperatively (Table 1).

**Table 1:** Demographic, diagnostic, and perioperative characteristics.

Characteristic	Value
Gender (male/female, *n*)	11/18
Age (years)	60.2 ± 7.5
BMI (kg/m²)	25.8 ± 3.1
Diagnosis, *n* (%)	
Lumbar spinal stenosis with disc herniation	15 (51.7)
Lumbar spondylolisthesis with disc herniation	12 (41.4)
Degenerative scoliosis with disc herniation	2 (6.9)
Surgical levels, *n* (%)	
Single-level	27 (93.1)
L2–L3	4
L3–L4	7
L4–L5	16
Two-level (L3–L5)	2 (6.9)
Clinical outcomes of Single-level Endo-OLIF	
Estimated blood loss (mL)	108.7 ± 32.4
Total operative time (min)	123.5 ± 18.6
Endoscopic decompression time (min)	58.2 ± 10.3
Length of hospital stay (days)	5.8 ± 1.7(range 3–10)

Data from the two patients who underwent two-level Endo-OLIF (*n* = 2) are presented separately in Table 4.

#### Clinical outcomes: pain and functional improvement

Significant improvements in pain and functional status were observed after Endo-OLIF and were maintained throughout the follow-up period (Table 2).

**Pain reduction:** The mean VAS score for back pain decreased from 7.1 ± 1.3 preoperatively to 2.2 ± 0.8 at 3 months postoperatively (*P* < 0.001) and further improved to 1.6 ± 0.7 at the final follow-up (*P* < 0.001 compared with preoperative; *P* > 0.05 compared with 3-month). Similarly, the mean VAS score for leg pain decreased from 6.3 ± 1.2 preoperatively to 1.8 ± 0.6 at 3 months (*P* < 0.001) and to 1.3 ± 0.5 at the final follow-up (*P* < 0.001). At the final follow-up, 26 patients (89.7%) reported complete or near-complete resolution of leg pain (VAS ≤ 2), and 24 patients (82.8%) reported satisfactory relief of back pain. For VAS, the minimum clinically important difference (MCID) was defined as a reduction of ≥ 1.5 points for back pain and ≥ 1.8 points for leg pain, based on established thresholds (13, 14). By these criteria, 26 patients (89.7%) achieved MCID for back pain VAS and 27 patients (93.1%) achieved MCID for leg pain VAS.
**Functional improvement:** The mean ODI score improved dramatically from 66.8 ± 9.7 preoperatively, indicating severe disability, to 17.5 ± 8.2 at 3 months postoperatively (*P* < 0.001), corresponding to mild to moderate disability. At the final follow-up, the ODI had further improved to 12.6 ± 7.1 (*P* < 0.001). A total of 25 patients (86.2%) achieved a minimum clinically important difference (MCID) for ODI (defined as a reduction of ≥ 12.8 points), and 22 patients (75.9%) achieved excellent outcomes (ODI < 10).

**Table 2:** Pain and functional scores at different time points.

Score index	Preoperative	3 months postoperative	Last follow-up	*P*-value
Back pain VAS	7.1 ± 1.3	2.2 ± 0.8*	1.6 ± 0.7*	< 0.001
Leg pain VAS	6.3 ± 1.2	1.8 ± 0.6*	1.3 ± 0.5*	< 0.001
ODI	66.8 ± 9.7	17.5 ± 8.2*	12.6 ± 7.1*	< 0.001

VAS, visual analogue scale; ODI, Oswestry disability index.

*P < 0.001 compared with preoperative values. No significant difference was observed between 3-month and final follow-up values (*P* > 0.05).

MCID for ODI was achieved in 86.2% of patients. MCID for VAS (back pain ≥ 1.5, leg pain ≥ 1.8) was achieved in 89.7% and 93.1% of patients, respectively.

Notably, no significant differences were observed between the 3-month and final follow-up assessments for any clinical outcome measure (*P* > 0.05 for all), indicating that the clinical benefits achieved early postoperatively were well sustained over time.

#### Radiological outcomes: decompression and restoration of spinal alignment

All radiological parameters demonstrated significant improvements from preoperative to postoperative assessments, with no substantial loss of correction at final follow-up (Table 3).

**Figure 2:**
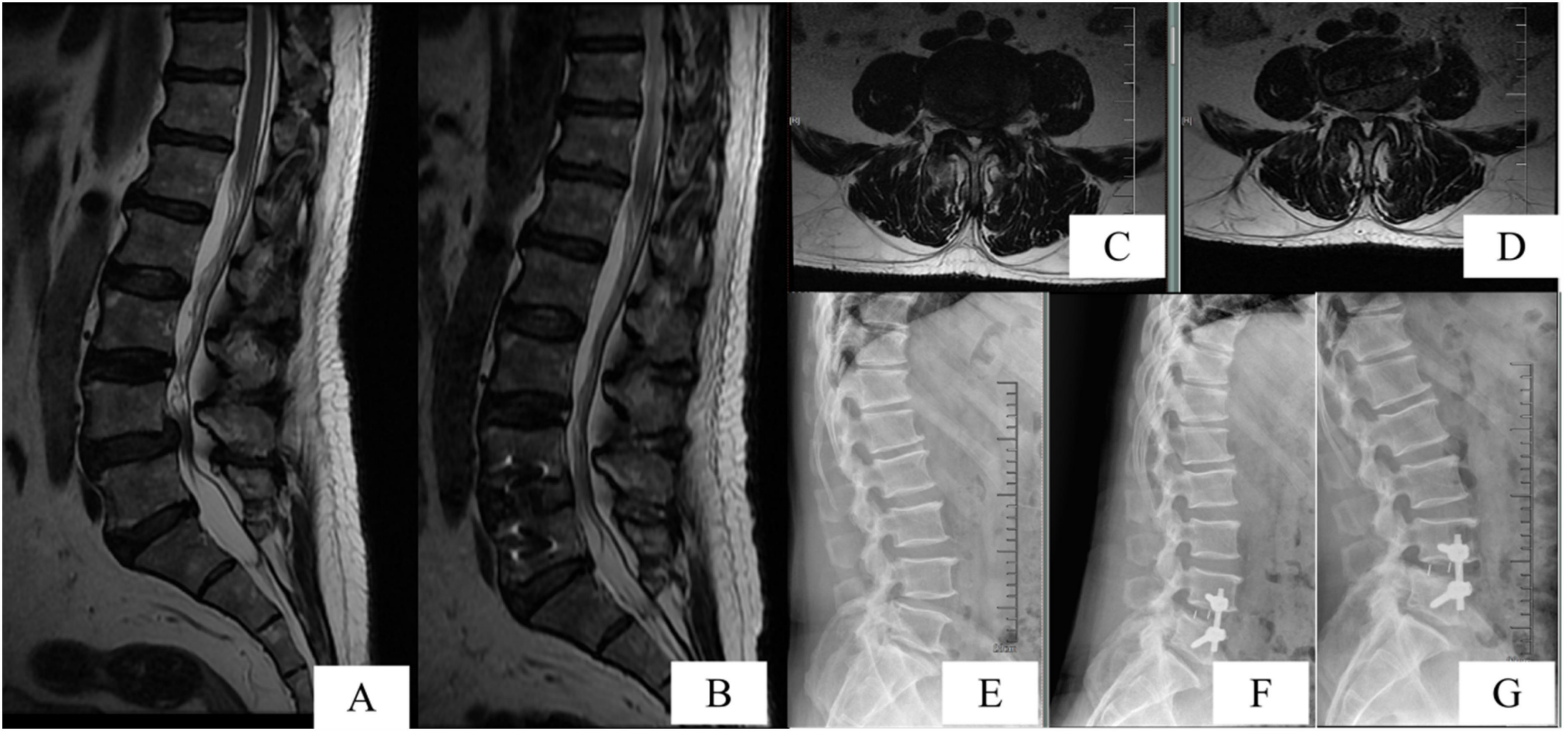
Clinical imaging data of a 68-year-old male patient with L4-L5 disc herniation and spinal stenosis. Preoperative MRI **(A,C)** showed severe canal stenosis. Postoperative MRI **(B,D)** demonstrated complete decompression after Endo-OLIF. The patient's VAS scores improved from 6 (back) and 9 (leg) preoperatively to 1 and 0 postoperatively. Radiographs at 3 months and final follow-up **(E–G)** showed maintained disc height and stable screw positioning without loosening or cage subsidence.

**Disc height restoration:** The mean disc height increased from 6.2 ± 1.5 mm preoperatively to 13.8 ± 1.9 mm immediately postoperatively (*P* < 0.001), representing a mean increase of 122.6%. At the final follow-up, disc height was well maintained at 12.5 ± 1.8 mm, with a mean loss of only 1.3 mm (9.4%) from the immediate postoperative value, indicating satisfactory maintenance of disc space distraction.
**Lordosis correction:** The segmental lordosis angle improved significantly from 10.5° ± 4.2° preoperatively to 19.8° ± 2.7° postoperatively (*P* < 0.001) and remained stable at 18.2° ± 2.5° at final follow-up. The global lumbar lordosis angle increased from 33.6° ± 8.3° to 42.1° ± 5.6° postoperatively (*P* < 0.001) and was 40.3° ± 5.1° at final follow-up. These improvements indicate that Endo-OLIF effectively restores both segmental and global sagittal alignment.
**Neural decompression:** The mean spinal canal area expanded from 73.2 ± 13.8 mm² preoperatively to 136.5 ± 21.4 mm² postoperatively (*P* < 0.001), an increase of 86.5%. At final follow-up, the spinal canal area remained significantly enlarged at 124.8 ± 20.7 mm². The mean foraminal area increased from 70.5 ± 7.2 mm² preoperatively to 143.2 ± 9.8 mm² postoperatively (*P* < 0.001), a 103.1% increase, and was 134.6 ± 10.5 mm² at final follow-up. Postoperative MRI in all patients confirmed complete removal of herniated disc material and adequate decompression of the dural sac and exiting nerve roots without evidence of residual compression (Figure 2).
**Brief note on two-level cases:** The two patients who underwent two-level Endo-OLIF for degenerative scoliosis-associated disc herniation showed clinical improvements comparable to the single-level group. Their detailed clinical and perioperative data are presented in Table 4. Both patients achieved satisfactory fusion without complications. However, the small sample size (*n* = 2) precludes formal statistical comparison with the single-level cohort.

**Table 3:** Radiological parameters at different time points.

Radiological index	Preoperative	3 months postoperative	Last follow-up	*P*-value
Disc height (mm)	6.2 ± 1.5	13.8 ± 1.9*	12.5 ± 1.8*	< 0.001
Segmental lordosis angle (°)	10.5 ± 4.2	19.8 ± 2.7*	18.2 ± 2.5*	< 0.001
Lumbar lordosis angle (°)	33.6 ± 8.3	42.1 ± 5.6*	40.3 ± 5.1*	< 0.001
Spinal canal area (mm²)	73.2 ± 13.8	136.5 ± 21.4*	124.8 ± 20.7*	< 0.001
Foraminal area (mm²)	70.5 ± 7.2	143.2 ± 9.8*	134.6 ± 10.5*	< 0.001

*P < 0.001 compared with preoperative values. No significant loss of correction was observed between postoperative and final follow-up assessments (*P* > 0.05 for all parameters).

**Table 4:** Clinical and perioperative data of the two patients who underwent two-level Endo-OLIF (L3–L5).

Parameter	Patient 1	Patient 2
Age (years)/Sex	68/Female	71/Male
Diagnosis	Degenerative scoliosis with disc herniation	Degenerative scoliosis with disc herniation
Surgical levels	L3–L5	L3–L5
Operative time (min)	165	160
Estimated blood loss (mL)	150	120
Length of hospital stay (days)	6	5
Clinical outcomes		
Back pain VAS (preoperative → final)	7 → 1	8 → 2
Leg pain VAS (preoperative → final)	6 → 1	6 → 1
ODI (preoperative → final)	69 → 13	68 → 15
Fusion status	Satisfactory	Satisfactory
Complications	None	None

Only two patients underwent two-level procedures in this series; therefore, individual data are presented without descriptive statistics, and formal statistical comparison with the single-level group is precluded.

### Complications and adverse events

No major intraoperative complications, including dural tear, nerve root injury, vascular injury, or retroperitoneal hematoma, occurred in this series. Postoperatively, two patients (6.9%) experienced transient ipsilateral thigh numbness or hip flexion weakness, which we attribute to intraoperative retraction and blunt stimulation of the lumbar plexus, both resolved completely within 6 weeks without specific intervention. One patient (3.4%), who had pre-existing diabetes mellitus, developed incisional fat liquefaction with poor wound healing. The wound was managed with regular dressing changes and meticulous glycemic control, and eventually healed completely without surgical debridement or systemic antibiotic therapy. No patient required revision surgery for cage migration, screw loosening, or recurrent herniation during the follow-up period. There were no cases of symptomatic adjacent segment degeneration or new-onset instability.

## Discussion

The anterior-to-psoas surgical corridor for lumbar interbody fusion was initially reported by Mayer in 1997 (15) and was subsequently named OLIF by Silvestre et al. in 2012 (4). OLIF has emerged as a cornerstone of minimally invasive spine surgery, offering distinct advantages over traditional posterior approaches such as PLIF and TLIF. Unlike PLIF and TLIF, which require extensive dissection of paraspinal muscles and posterior ligamentous structures, OLIF utilizes a retroperitoneal approach anterior to the psoas muscle, preserving the posterior tension band and reducing the risk of postoperative instability. This approach also allows for the insertion of larger interbody cages, which can better restore disc height, foraminal height, and lumbar lordosis compared with posterior approaches (16, 17).

The advantages of OLIF over other fusion techniques include reduced blood loss, shorter hospital stays, and lower rates of postoperative complications such as dural tears and nerve root injuries. Additionally, OLIF avoids the risk of lumbar plexus injury associated with direct lateral interbody fusion by maintaining a trajectory anterior to the psoas muscle. However, OLIF has limitations, particularly in cases requiring direct neural decompression. While OLIF provides indirect decompression through disc height restoration and ligamentotaxis, it is less effective in cases of large disc herniations, migrated disc fragments, or severe central canal stenosis. Fujibayashi et al. prospectively validated the efficacy of indirect decompression via OLIF, demonstrating a significant increase in dural sac cross-sectional area from 99.6 mm² preoperatively to 134.3 mm² postoperatively (*P* < 0.001), and identified preoperative dural sac area as the sole independent predictor of decompression efficacy (18). However, OLIF has inherent limitations in cases with large herniated discs or migrated fragments. Malham et al., in a prospective study of 122 patients who underwent lateral lumbar interbody fusion (LLIF), found that 11 patients (9.0%) required additional posterior surgery due to inadequate indirect decompression. Of these, 7 failures were attributed to insufficient preoperative evaluation of instability or bony stenosis, 3 to suboptimal cage placement, and 1 to unexplained causes (19). These findings underscore the importance of rigorous patient selection for indirect decompression and highlight the clinical value of combining endoscopy for direct neural decompression.

In the present cohort, the mean disc height increased by 122.6% postoperatively, and the spinal canal and foraminal areas expanded by 86.5% and 103.1%, respectively. These substantial morphological improvements directly translated into clinically meaningful pain relief and functional recovery, as evidenced by an 86.2% MCID achievement rate for ODI. Notably, the clinical benefits were sustained through the final follow-up, with no significant deterioration in VAS or ODI scores from 3 months postoperatively, underscoring the durability of Endo-OLIF.

**Figure 3:**
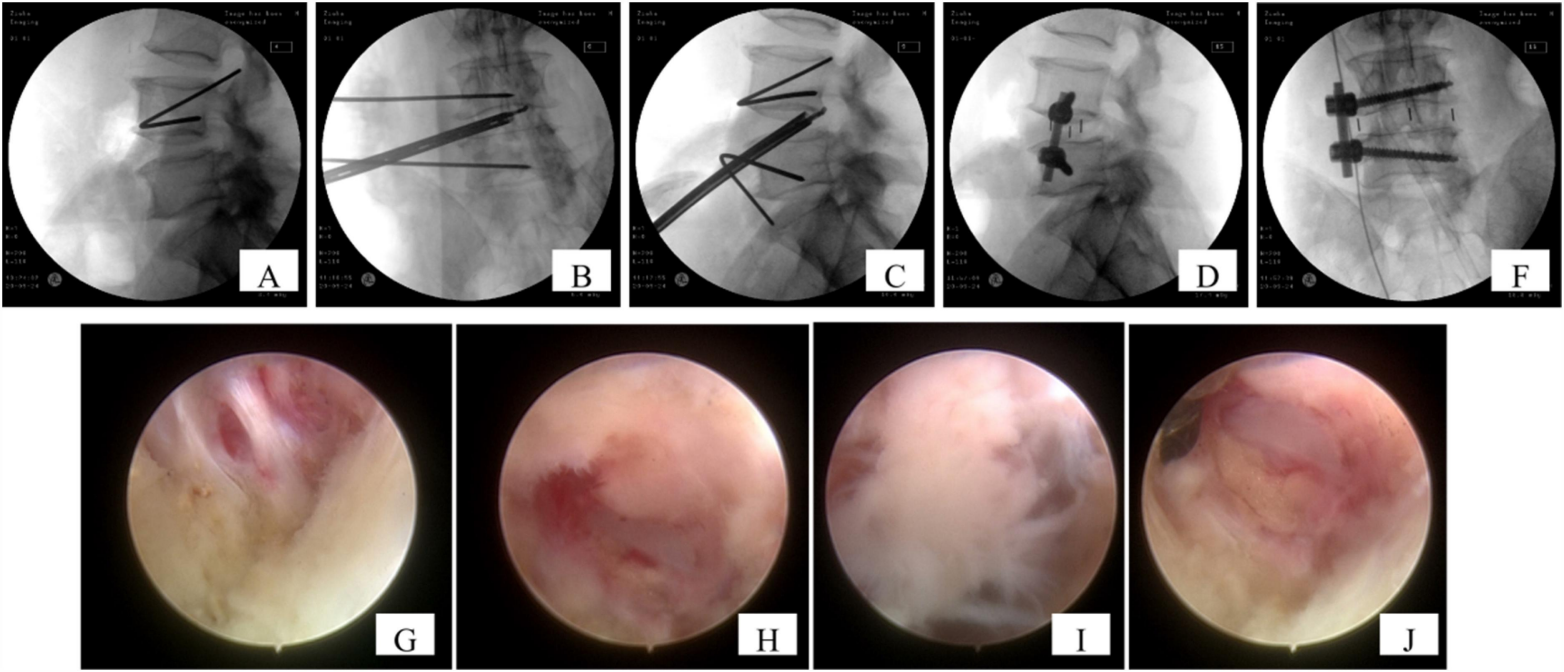
Intraoperative fluoroscopy and endoscopic decompression during Endo-OLIF. **(A,B)** Two guidewire pins placed on the superior and inferior vertebral bodies. **(C)** The radiofrequency probe smoothly accessing the target decompression site. **(D–F)** Lateral screw placement was performed under fluoroscopic guidance to enhance construct stability; this technique allowed screw insertion through the same incision without the need for additional posterior incisions, thereby avoiding iatrogenic damage to posterior lumbar paraspinal muscles, facet joints, and adjacent soft tissues. **(G)** Exposure of the posterior margin of the intervertebral disc space. **(H)** Partial resection of the posterior longitudinal ligament to expose the dural sac and herniated nucleus pulposus. **(I)** Removal of sequestered disc fragments. **(J)** Complete dural sac decompression achieved.

The advent of spinal endoscopy has revolutionized the treatment of LDH by enabling direct visualization and targeted decompression of neural structures (Figure 3). Full-endoscopic techniques, such as percutaneous endoscopic lumbar discectomy, allow for precise removal of herniated disc material with minimal tissue disruption (20). The magnified view provided by the endoscope enhances the surgeon's ability to identify and remove migrated disc fragments, even in challenging locations such as the epidural space or foraminal regions (21). Moreover, spinal endoscopy is associated with reduced postoperative pain, faster recovery, and lower complication rates compared with open surgical techniques (22).

The integration of spinal endoscopy into lumbar fusion procedures has further expanded its applications. Endoscopic-assisted techniques enable direct decompression of neural elements while preserving posterior structures, making them particularly useful in cases where indirect decompression alone is insufficient. Heo et al. demonstrated that endoscopic-assisted OLIF could effectively remove central and foraminal disc herniations, achieving both direct and indirect decompression in a single procedure (23). This combined approach, which we refer to as Endo-OLIF, leverages the strengths of both techniques: OLIF provides structural stability and indirect decompression, while spinal endoscopy ensures direct neural decompression under magnified visualization.

In this study, the integration of spinal endoscopy into the OLIF procedure allowed for complete removal of herniated disc material, as confirmed by postoperative MRI. The mean endoscopic decompression time was 58.2 ± 10.3 min, and the procedure was associated with low intraoperative blood loss (108.7 ± 32.4 mL) and a short hospital stay (5.8 ± 1.7 days). The endoscopic view facilitated the removal of migrated disc fragments, even those located in the epidural space or foraminal regions, which are often challenging to access through traditional OLIF alone. Additionally, the use of endoscopic visualization ensured thorough endplate preparation, reducing the risk of incomplete fusion.

Our results also provide preliminary evidence that Endo-OLIF may be feasible for carefully selected patients with two-level degenerative disease, although the small number (*n* = 2) of two-level cases in this series precludes definitive conclusions. Both patients with degenerative scoliosis-associated disc herniation achieved good clinical outcomes without increased complications, suggesting that the hybrid technique could be extended beyond single-level pathology. Nevertheless, larger comparative studies are needed to establish the risk–benefit profile of multilevel Endo-OLIF.

While lateral screw fixation is commonly used to enhance the stability of OLIF constructs, its efficacy and safety remain subjects of debate. Proponents argue that lateral screws reduce operative time, intraoperative bleeding, radiation exposure, and soft tissue trauma, enabling one-stage interbody fusion with a single incision (24, 25). However, opponents contend that lateral screw placement, given its proximity to critical neurovascular structures, may increase the risk of vascular injury and nerve root irritation. Additionally, lateral screws have been associated with higher rates of cage subsidence and screw loosening (26, 27). In this study, all patients underwent lateral screw fixation, and no complications related to screw placement were observed. Nevertheless, the optimal fixation strategy for OLIF remains unclear, and further research is needed to evaluate the long-term outcomes of lateral screw fixation compared with alternative techniques such as posterior pedicle screw fixation.

Regarding postoperative wound complications, one diabetic patient developed incisional fat liquefaction and delayed healing, which was successfully managed with conservative measures. This case highlights the importance of preoperative optimization of glycemic control and meticulous soft tissue handling during the retroperitoneal approach, particularly in patients with metabolic risk factors. Notably, no surgical site infection requiring antibiotics or debridement occurred, suggesting that Endo-OLIF does not inherently increase infection risk compared with other minimally invasive fusion techniques.

It should be acknowledged that Endo-OLIF is a technically demanding procedure with a substantial learning curve. The surgeon must be proficient in both the retroperitoneal approach for OLIF and full-endoscopic decompression techniques. Based on our institutional experience, we recommend that surgeons interested in adopting this technique first gain competence in OLIF and spinal endoscopy separately before attempting the combined procedure. Proctoring by experienced surgeons and careful patient selection during the initial learning phase are strongly advised. In our series, the operative time decreased progressively after the initial 10 cases, suggesting a learning curve of approximately 10–15 cases to achieve procedural efficiency. Future studies with larger cohorts may further define the learning curve characteristics of Endo-OLIF.

While no vascular injuries occurred in this series, the proximity of the retroperitoneal approach to major abdominal vessels—including the abdominal aorta, inferior vena cava, and iliac vessels—entails an inherent risk of vascular complications. The addition of endoscopic decompression within this corridor further increases the complexity of the procedure. Therefore, we emphasize that Endo-OLIF should be performed only in institutions with immediate access to vascular surgical expertise. A close collaborative relationship with vascular surgeons is essential for preoperative planning and for the management of potential vascular injuries. This technical requirement may limit the widespread applicability of Endo-OLIF to well-organized medical centers with multidisciplinary support.

Furthermore, given the technical complexity and potential risks associated with Endo-OLIF, we emphasize that this technique should be reserved for carefully selected patients with complex degenerative lumbar pathologies requiring both structural stabilization and direct neural decompression. It is not recommended for routine or uncomplicated degenerative conditions that can be adequately managed by simpler techniques. The procedure is best performed in high-volume centers with appropriate multidisciplinary support, including vascular surgery backup, and by surgeons who have completed the requisite learning curve.

Several limitations of this study should be acknowledged. First, patients with left-sided disc herniation or predominant left-sided spinal stenosis were excluded due to technical limitations of the left-sided OLIF corridor. Specifically, the trajectory of the working channel through the left-sided retroperitoneal approach creates an angular blind spot for left subarticular lesions, making complete endoscopic decompression technically challenging. Therefore, to ensure the safety and efficacy of the combined procedure, we restricted this initial series to patients with central, right-sided, or bilateral pathology in whom the endoscopic working angle was favorable for complete decompression. Future studies may explore modified endoscopic techniques or contralateral approaches to extend the indications of Endo-OLIF to left-sided pathologies. Second, the mean follow-up period of 15.6 months precludes assessment of long-term outcomes, particularly adjacent segment degeneration rates. Third, the single-center design introduces potential selection bias, although this was mitigated by consecutive enrollment protocols. Fourth, the sample size of 29 patients is relatively small, which may limit the generalizability of our findings. Fifth, the inclusion of only two patients with degenerative scoliosis limits the generalizability of our findings to this specific subgroup. Sixth, while we observed promising outcomes in two-level procedures, the sample size is too small to support routine clinical adoption of multilevel Endo-OLIF. Future multicenter prospective studies with larger sample sizes and longer follow-up durations are warranted to validate these preliminary results.

## Conclusion

In conclusion, Endo-OLIF represents a significant advancement in minimally invasive spine surgery. Our results corroborate growing evidence that this combined approach safely expands OLIF indications while preserving its inherent advantages. However, it should be recognized that Endo-OLIF is a technically demanding procedure with an inherent risk of vascular injury due to the proximity of major abdominal vessels. Therefore, it should be performed only in well-organized medical centers with multidisciplinary support, including vascular surgical expertise, and by surgeons who have completed the requisite learning curve. As the field evolves, continued refinement of surgical protocols, coupled with advanced navigation technologies, will likely establish this technique as a first-line option for complex degenerative pathologies requiring both structural stabilization and direct neural decompression.

## Data Availability

The raw data supporting the conclusions of this article will be made available by the authors, without undue reservation.
